# Policy as soft deterrence: Impact of recent policy changes on international students in Australia

**DOI:** 10.1177/0193841X251405523

**Published:** 2025-12-11

**Authors:** Ly Thi Tran, Trang Le, Jill Blackmore, Baogang He, Huy Quan Vu

**Affiliations:** 1 School of Education, 2104 Deakin University, Burwood, Victoria, Australia; 2 School of Humanities and Social Sciences, Deakin University, Burwood, Victoria, Australia; 3 Deakin Business School, 2104 Deakin University, Burwood, Victoria, Australia

**Keywords:** international education, international students, impact, educational policy, policy analysis, affective governance, student experiences, temporal bordering, aspirational compromise

## Abstract

Since 2021, Australia’s international education and migration policies have undergone significant changes. However, a critical gap remains in understanding how these policy shifts are experienced, interpreted, and evaluated by international students themselves. Drawing on in-depth interviews with Chinese, Indian and Vietnamese international students, this article examines how these cohorts make sense of and navigate the evolving landscape of Australia’s international education policies. It unpacks their experiences of administrative delays, visa insecurity and escalating visa fees, constrained employment opportunities, and emotional uncertainty, and shows how these vary across national backgrounds. By centring student voices, the analysis moves beyond official policy rhetoric to explore how international education and migration governance is lived, evaluated, and internalised in students’ everyday life. In doing so, the article contributes to a deeper understanding of how seemingly technical policy instruments function as technologies of affect, shaping not only international education and migration outcomes but also students’ sense of belonging, self-worth, and future possibilities. Particularly, this article offers an original conceptual framework by introducing three new concepts to the international education literature: ‘temporal bordering’, ‘aspirational compromise’, and ‘affective governance in international education’ to illuminate how international education-migration policies shape international students’ experiences, aspirations, emotions, and sense of belonging. It reconceptualises international education governance as relational and affective, moving beyond macro-level policy analysis to highlight the affective dimensions of students’ experiences with shifting geopolitical and policy contexts.

## Introduction

Australia’s international education sector has experienced significant policy shifts in recent years. Once recognised as a global leader that combined quality education with attractive post-study work and migration opportunities, Australia has increasingly adopted restrictive measures that are reshaping international student mobility. As the country’s largest services export, international education contributed over $51 billion to the economy in 2024 (Department of Education, 2024) and enriches Australia’s educational, cultural and diplomatic relations. Decades of declining investment in research (from 2.2% to 1.7% of GDP) have left Australian universities increasingly reliant on international student fees to fund research and to support the teaching and learning of domestic students. This dependence poses significant risks, especially for mid- and lower-tier institutions (Blackmore, 2023). These ongoing policy shifts have intensified both public and academic scrutiny of Australia’s precarious balancing act – managing economic dependence on international students, implementing more stringent controls, and navigating the politicisation of international education amidst rising inward-looking nationalism and populist political narratives (Tran et al., 2023).

Policy reforms implemented since 2021, including the Migration Strategy 2023, risk-tiered visa systems, the Genuine Student Test, restricted post-study work rights, and increased visa fees, have reconfigured international students’ migration trajectories and settlement aspirations. While these reforms are officially presented as measures to uphold the integrity of the international education sector and regulate migration flows (Fenton-Smith & Gurney, 2025), they have sparked considerable public debate and are perceived to have detrimental impacts on both education institutions and international students, with some universities laying off academics (Liu et al., 2023). Despite this, there remains limited understanding of how international students themselves perceive and navigate the scale, speed, and unpredictability of these changes. This article seeks to address this gap by examining students’ lived experiences of policy changes, manifested in administrative delays, visa precarity, restricted employment opportunities, and emotional uncertainty. Particular attention is given to variations across national cohorts, with a focus on students from China, India, and Vietnam – Australia’s key source countries for international education. As of June 2025, Australia hosted 169,950 Chinese students (23%), 126,172 Indian students (17%), and 33,725 Vietnamese students (5%), making these countries the first, second, and fourth largest sources of international students, respectively (Department of Education, 2025).

Drawing on Bacchi and Goodwin’s (2016) conceptualisation of policy as governmentality, alongside affect theory (Sedgwick, 2003), this research shows how recent policy reforms are evaluated and interpreted as government’s use of technologies of soft deterrence. We define ‘soft deterrence’ as the subtle shaping of behaviour and decision-making through administrative and regulatory practices rather than overt enforcement. While hard deterrence relies on force, threats, or direct exclusion, soft deterrence works indirectly through cultivated uncertainty, policy volatility, bureaucratic delay, and emotional regulation to discourage particular actions. In the Australian visa regime, it aims to deter applications from ‘undesirable’ or ‘risky’ student groups and discipline those already within the system. This aligns with Bacchi and Goodwin’s (2016) governmentality framework, which highlights how governance operates through everyday bureaucratic practices to guide and regulate individuals’ actions. Yet, soft deterrence is not merely technical; it is increasingly racialised and geopolitical, producing hierarchies of international students (e.g. privileging applicants from ‘trusted’ countries while constraining those from ‘risky’ ones) and embedding structural inequality into the infrastructure of education governance. The findings illustrate how these forms of emotional governance, particularly through visa precarity, opaque communication, and heightened scrutiny, foster an internalised sense of illegitimacy among international students. These policies subtly communicate differentiated worth, delineating who is perceived as desirable and welcome, and who is instead positioned as a suspect subject to heightened scrutiny, bureaucratic delays, and potential exclusion. The risk-tiered visa system functions as a mechanism of soft deterrence, exerting control through the cultivation of insecurity, the gradual erosion of belonging, and governance techniques manifested in strategic delays and bureaucratic opacity. What emerges is a visa regime increasingly shaped by racialised and geopolitical considerations, undermining the focus on transparency and merit-based criteria and privileging selective inclusion and exclusion.

This article offers a distinct conceptual contribution by introducing the concepts of ‘***temporal bordering***’, ‘***aspirational compromise***’, and ‘***affective governance in international education***’. ‘Temporal bordering’ describes how policy such as age caps and reduced post-study work rights durations operate to influence international students as temporary migrants’ future planning, constrain aspirations, and cultivate a persistent state of temporality and uncertainty (Robertson, 2019). The related concept of ‘aspirational compromise’ captures how international students are often compelled to compromise their educational goals or preferred study destinations in favour of perceived advantages in migration outcomes in response to policy volatility and tightened migration context. ‘Affective governance’ refers to how policies shape individual students’ feelings of legitimacy, belonging, and inclusion. In the context of shifting geopolitics, these concepts reposition international education and migration governance as affective, relational, and deeply stratified, contributing a nuanced, empirically grounded conceptualisation that moves beyond traditional administrative or macro-level policy analysis.

Enriched by these concepts, the study elucidates how geopolitics, risk logics, and commodification converge in the lived realities of student migration (Mezzadra & Neilson, 2012). International students may be viewed as calculated risk-takers, navigating their choices through a logic informed by aspirations of hope as well as trepidation. Beck (1992) argues that in the contemporary global risk society, perceptions of risk and decisions arising are informed by cultural beliefs as well as emotions of fear and desire. According to Bialostok (2015), ‘risk frames identities, shapes social relations and institutions, and extends decision-making and other forms of power to some groups while excluding these things from others’ (p. 561). Nation states in the current rapidly changing geopolitical context are becoming more risk averse as international students are now overtly associated with other priorities of governmentality such as national security and domestic politics.

## **The Evolving P** **olicy Landscape**

In response to rising anti-migration sentiment and domestic concerns over security, employment, and housing shortages, major destination countries have introduced increasingly restrictive and hostile policies that significantly impact international students and higher education institutions. In the United States, the Trump administration’s visa restrictions have drawn widespread criticism from universities. In April 2025, around 1,800 students and 280 institutions were affected by visa cancellations – later reversed – targeting those linked to pro-Palestinian protests, prior arrests, or politically sensitive online content (NBC News, 2025). Visa appointment scheduling was suspended during the peak season from May 27 to June 26, resulting in severe delays. Canada has also moved to curb temporary migration, extending a cap on international student enrolments through 2025 and 2026 due to pressure on housing and jobs (ICEF Monitor, 2024). This, along with new work permit restrictions and processing delays, is expected to cause a drop of over 50% in new international student arrivals compared to 2024 levels (The PIE News, 2025). In the United Kingdom, a government white paper released in May 2025 outlines plans to reduce migration across all visa categories, including student routes. The Graduate Route will be shortened from 24 to 18 months for those completing undergraduate and master’s degrees (UK government, 2025). The paper also signals tighter compliance requirements for UK institutions and the potential introduction of a levy on international student fees.

In Australia, international education strategy has shifted significantly over the years, influenced by evolving economic, political, and social forces. Key dimensions of international education, including inbound and outbound mobilities, transnational education and research partnerships, and internationalisation at home, have been linked more closely to economic, social, and geopolitical interests (Gribble & Tran, 2016; Tran, Bui et al., 2025). In the earlier phases, international education was aligned more with aid-focused efforts, human capability building and institutional, country and regional relation building. However, international education is increasingly seen as a mechanism for countries to exert their soft power and strengthen their regional or global position (Altbach & de Wit, 2018). Over time, international education policy has shifted from aid-driven approaches towards commercialisation, while also broadening to encompass public diplomacy and geopolitical aims alongside continuing priorities of collaboration and capability building (Tran & Bui, 2021; Tran & Nguyen, 2023; Tran, Nguyen et al., 2025; Tran Nguyen et al., 2023).

Until the early 1980s, international education was shaped by a dual purpose of providing aid and advancing soft power. Programs such as Australia’s Colombo Plan sponsored Asian scholars to study in Australia between 1951 and 1985. The U.S. Fulbright Program, supporting educational and cultural exchange between the United States and other countries mainly through mobility, aimed to build capacity and transfer knowledge to developing nations while also serving geopolitical interests by allowing donor countries to influence recipient states and attract global talent (de Wit, 2013). The shift of international education from aid to a commercial export industry aligned with the 1988 Dawkins reforms and the implementation of the Overseas Student Policy (Meiras, 2004), which corporatised higher education and introduced market-driven, managerial policies (Blackmore, 2023). This shift was fuelled by reduced public funding and growing demand for overseas education from Asia’s middle class, leading to a business-oriented model that boosted the recruitment of international students (Tran, 2013). With the shift from an aid-focused approach to a market-oriented model, international education in Australia has become increasingly commercialised. This trend is evident in recent policy frameworks such as the Australian Strategy for International Education 2021–2030, which emphasises market-driven objectives, alignment with workforce needs, and the generation of economic returns from international students (Fenton-Smith & Gurney, 2025). The commercialisation of the sector is reflected in policy measures such as enrolment caps, visa reforms, and targeted incentives for regional study, which together illustrate how international education is treated as an economic commodity, a political instrument and a mechanism to address national workforce demands. This shift from politically motivated to economically driven policies mirrors broader transformations in Australia’s political economy and highlights the tensions between educational, social, and market imperatives (Rhoades & Smart, 2018).

However, since 2020, international education has become increasingly politicised again with political motives underpinning recent policy changes in international education. Rising concerns are evident globally with the shifting geopolitical landscape as China, India, and others are asserting a multi-polar rather than Ameri-centric world order (Marginson, 2024a). This has caused tensions between educational institutions recruiting international students and government issuing policy changes. Institutional responses to policy changes are motivated both by economic priorities and international student welfare towards the survival and sustainability of international education and their own institutions while governmental rationalities related primarily to geopolitical priorities. The government’s unprecedented intervention in capping international student numbers reflects not only long-standing concerns about the reliance on international students but also a short-term political strategy tied to the 2025 federal election in which housing shortages and net migration were overtly linked to international students (Tran & Tan, 2024). This approach reveals a politically driven, micro-managed effort to reduce Net Overseas Migration by using international education as a scapegoat.

Since 2021, Australia’s international education sector has experienced major policy changes, shifting from a focus on growth and accessibility to tighter regulations and enrolment caps, serving political interests and workforce needs. Key workforce shortages are in teaching, nursing, and engineering whereas a large proportion of international students enrol in business studies. The first significant shift in policy was the Australian Strategy for International Education 2021–2030 launched in November 2021. This strategy aimed to rebuild the sector sustainably, focussing on four key priorities that realigned international education with national interests. First, the aim was to reduce the over reliance on China and India as major sources by diversification of source countries. Second, the aim was to ensure educational offerings meet national employment demands align with workforce and skills needs. Third, putting students at the centre required improving their experiences, thereby increasing Australia’s attractiveness, and finally, enhancing Australia’s position in the global education market was aimed for through growth and improved competitiveness.

Three years later, the further rapid shift in geopolitics and the impact of lack of regulation on visas during and after COVID-19 by the Coalition government had led to rapid escalation of student numbers (Beatty, 2025). This was attributed to the extension of student work rights and post-study work rights. Visa delays were exacerbated by incomplete applications and heightened scrutiny over student visa integrity (Ross, 2024). These delays were also driven by increased visa vetting due to concerns about non-genuine applicants and the rise of so-called ‘visa factories’ or ‘ghost colleges’, prompting the Department of Home Affairs to apply stricter checks and documentation requirements. There were rising concerns that many students from some source countries (e.g. India and Nepal) sought to gain work rather than study due to visa loopholes. In July 2024, the Australian Government introduced a number of policy changes that created further restrictions on students and universities. These included raising the fee for international student visas from $710 to $1,600 (increased to $2,000 in July, 2025); limiting of the Temporary Graduate visa, including age restrictions and reduced post-study work rights (PSWR); placing an indicative enrolment cap of 270,000 for new international student enrolments with 145,000 allocated to university enrolments, and finally, individual caps were set for each Australian university (Australian Government, 2024).

The Labour government argued that the changes aimed to ensure controlled and sustainable management of Australia’s international education sector, integrity of the sector, and balancing economic benefits with migration considerations and skills demands. Key stakeholders including education providers, peak bodies of international education, and some state/territorial and local governments contended that reducing international student numbers could not only damage Australia’s international education standing but also hinder Australia’s economic growth, innovation, and productivity. For example, the Victorian Government warned that the enrolment caps could cost the state’s economy $5.9 billion and 12,000 jobs by 2027 (Victorian Government, 2024). The proposed caps also jeopardised the state’s capacity to tackle existing local skill shortages.

Universities have expressed concern that these policy changes may jeopardise revenue for research and teaching, result in job losses, and diminish Australia’s prosperity (Universities Australia, 2025). In March 2024, the Australian government introduced the Genuine Student Test (GST), replacing the old Genuine Temporary Entrant (GTE) requirement for student visa (subclass 500) applications. Instead of a 300-word written statement, students now answer targeted questions that help visa officers understand their reasons for studying in Australia alongside the documents they submit. In July 2024, the introduction of a maximum age limit of 35, down from 50, for post-study temporary graduate visa applicants, except for higher degree research students, has significantly reduced demand for student visas. This policy shift led to a sharp decline in applications from 30 to 34-year-olds, many of whom would age out before completing their studies, particularly affecting migration-sensitive countries like India and Pakistan (Tran & Tan, 2024).

To tighten international student numbers, the government implemented three major shifts in direction over the past 2 years. Ministerial Direction 107, released by the Australian government in December 2023, established processing priorities for student and student guardian visa applications by prioritising applications based on the risk level of education providers and the applicant’s country of citizenship. It faced criticism for disproportionately affecting certain education providers, regional universities and students, leading to calls for its revocation (Martin, 2024). In response, Ministerial Direction 111 was introduced in December 2024 to delay visa processing for institutions approaching their enrolment limits, effectively acting as an informal cap without the need for formal legislation. This measure has resulted in a notable decline in student visa applications (‘Direction on Foreign Students’, 2025). Consequently, mid- and low-tier universities, many regional, such as Victoria University, Charles Sturt, Wollongong, and Southern Queensland, whose students largely came from India and Nepal have made staff redundant (Chau, 2024) while Chinese students have been fast tracked into the Group of eight research-intensive universities. In August 2025, the government announced the National Planning Level for 2026, setting a cap of 295,000 international student places, 25,000 more than 2025, with all existing providers retaining their current allocations. Direction 115, which came into effect on 14 November 2025, replaces Direction 111 and integrates the recent new overseas student commencements (NOSC) exemptions for public providers, including expanded purpose-built student accommodation, stronger Southeast Asia engagement, and transnational education delivery. It also imposes a tighter limit, restricting providers to 115% of their NOSC allocation, with any excess placing them in a Priority 3 visa processing category. The timeline to the policy changes since 2021 was summarised in Appendix 1

These policies have been regarded as the unprecedented intervention to international students, reflecting the weaponisation of international students, positioning international students as not only economic objects but also political tools and scapegoats. The frequent and unpredictable changes reflect an uncertain and volatile environment. The government has faced criticism, especially from the Group of Eight (2024), for prioritising short-term political gains over long-term national interests, including socio-economic and diplomatic objectives, and for demonstrating a perceived lack of respect and reciprocity toward international students and their home countries.

Ironically, the recent surge of policy changes in Australian international education has unfolded alongside national strategies such as the Draft International Education and Skills Strategic Framework, the Australian Strategy for International Education 2021–2030, and the Universities Accord (Australian Government, 2024), which emphasise improving international student experiences, incorporating student voices into policymaking, and encouraging enrolment in regional universities. Yet, in practice, discussions around these reforms have been dominated by government and institutional actors, with limited direct input from international students themselves. This disconnect reflects broader dynamics of soft deterrence and risk governance, where policy frameworks seek to manage student mobility while controlling institutional and national risk, and governmentality and affect, shaping student behaviours through regulatory mechanisms and affective appeals (Marginson, 2024a; Tran, Tan, Bui & Rahimi., 2025). To ensure reforms are both effective and equitable, student voices must be actively incorporated, particularly from key source countries such as China, India, and Vietnam. This article addresses this gap in current policy formulation and current debates about policy changes by reporting the perspectives of international students on these issues. The next section outlines the theoretical framework underpinning this study.

## Theoretical Framework

This article is underpinned by a theoretical lens combining Bacchi and Goodwin’s (2016) governmentality framework and affect theory (Sedgwick, 2003). Governmentality, drawing on Foucault, examines how power operates through policies, shaping subjects and social practices (Larsson, 2018). Key concepts include:

• **Problematisation:** How issues are framed as ‘problems’ requiring intervention.
• **Rationalities:** The underlying logics or justifications guiding governance.
• **Technologies of self:** Ways individuals internalise governance, regulating their own behaviour in line with policy expectations.

Bacchi’s ‘What’s the Problem Represented to Be?’ (WPR) approach provides a structured tool for analysing policy construction. Rather than focussing on problem-solving, WPR critically examines how problems are constructed, revealing assumptions, power dynamics, and institutional knowledge embedded in policy. The WPR framework guided our analysis of interview data using the following questions (adapted from Bacchi, 2016):

**Table:** 

WPR question	Analytical purpose
What’s the problem represented to be?	Identifies how the policy frames the ‘problem’
What presuppositions or assumptions underlie this representation?	Uncovers taken-for-granted beliefs shaping policy
How has this representation come about?	Explores political, historical, social, and cultural origins
What is left unproblematic?	Reveals silences, exclusions, and neglected dimensions
What effects are produced by this representation?	Examines impacts on subjects, institutions, and social relations
How/where could this representation be questioned or disrupted?	Supports critical reflection and alternative framings

Applying WPR allowed us to systematically interrogate policies shaping international students’ experiences, highlighting both structural power relations and affective dimensions. Governmentality clarifies how power operates, while affect theory illuminates the emotional consequences of these governmental techniques on subjects.

The turn to affect in areas such as cultural studies, sociology, and education sheds light on how power is exercised, how social structures and policy representation shape people’s emotions, experiences, and a sense of inclusion/exclusion and thus how policies are experienced (Sedgwick, 2003; Tedeschi & Gadd, 2021; Waerniers & Hustinx, 2020). Affect theory brings together mind and body, emotion, and reason, enabling researchers to unpack feelings of shame, happiness, and paranoia (Sedgwick, 2003). Affects are intensities that move us. Affective environments foreground how emotions such as hope, frustration, or fear intersect with policy, shaping experiences of belonging, inclusion, or marginalisation (Sedgwick, 2003; Tedeschi & Gadd, 2021; Waerniers & Hustinx, 2020).

Affect theory has been used together with Bacchi and Goodwin’s (2016) governmentality framework to elucidate the ways in which policy is represented, the affective governance embedded in policy representation and its emotional impacts informing individual choices. Within the context of policy and governance, affect theory highlights how policies and modes of governance create affective environments in which individual emotions shape and are shaped by behaviour and power relations and in turn how hope, frustration, and fear inform feelings of belonging and marginalisation (Waerniers & Hustinx, 2020). Affect theory therefore provides an analytical lens for understanding the ways in which emotions and embodied experiences influence but at the same time governs social and political structures. Emotional reactions to policy should not be understood solely as individual experiences; rather, they are infused with political meaning and reasoning, playing a critical role in the construction of subjectivities and in either reinforcing or contesting dominant ideological structures. For international students, affective dimensions can intersect with a spatio-temporal approach in the ways that the emotional geographies of migrants are shaped by interconnected dimensions of space and time, revealing how past experiences of hope and fear continue to influence their present-day navigation of everyday life, activities, and relationships (Tedeschi & Gadd, 2021).

Within the international education settings, existing literature has extensively documented the economisation of international student identities (Lomer, 2018). However, recent scholarship identifies emerging discourses that recognise students as potential citizens and affective subjects with complex motivations and experiences (Tran, Tan et al., 2023; Hong et al., 2022). These shifting framings reflect broader tensions between neoliberal approaches to education, soft power diplomacy objectives, and rising ethnonationalist sentiments in host nations (Liu et al., 2023). Within this evolving landscape, students’ education and migration trajectories are shaped not only by their individual choices and institutional connections but also by their capacity to navigate structural precarity embedded in policy design (Robertson, 2019). Within this process of navigation, international students’ and migrants’ emotional responses to policy design and implementation play a critical role in shaping their sense of belonging, inclusion, marginalisation, and perceived worth (Lee et al., 2024; Tran, Bui et al., 2025). This article explores the affective impacts of policy changes, aiming to illuminate not only how policies influence international students’ lived experiences, emotions, aspirations, and future trajectories but also how such policies are embodied and experienced in their everyday lives.

## Research Design and Methodology

This article emerges from a broader research project funded by the Australian Research Council examining the geopolitics of student mobility and international education. The study includes on data from interviews with key stakeholders (students, university executives, researchers, and policy makers) in the international education sector, alongside national surveys and interviews with international students from China, India, and Vietnam studying in Australia, as well as Australian students studying abroad in those three countries. These student groups were selected as they represent three of Australia’s top five countries for both inbound and outbound student mobility (Department of Education, 2025). In addition, these three cohorts represent distinct risk categorisations and study-migration pathways, allowing for a more nuanced analysis of policy impacts. This article specifically focuses on qualitative in-depth interview data collected from 30 international students from China, India, and Vietnam. In-depth interviews allow for nuanced exploration of participants’ lived experiences and emotions, providing the depth and flexibility (Lincoln & Guba, 1985) needed to capture the affective dimensions of international students’ experiences. This method also enables participants to articulate their perspectives in their own terms, which is essential for understanding the complexities of their emotional experiences.

The recruitment criteria include that participants are (1) international students from China, India, and Vietnam, and (2) currently enrolled at an Australian university. Table 1 summarised the demographic information of the participants. Recruitment was carried out using purposive snowball sampling and through channels such as social media and professional networks, including LinkedIn. The researchers posted a call for participation outlining the recruitment criteria and inviting eligible students to participate. Snowball sampling then enabled existing participants to refer peers within their own communities, which was particularly useful for reaching students who might not be easily accessible through formal channels. These networks were instrumental in broadening the pool of participants. Ethics approval was obtained from the university where the researchers are based. Informed consent was obtained from all participants and measures were implemented to protect their anonymity, including the removal of identifying information such as names and institutional affiliations. Additional precautions were taken when reporting sensitive migration-related experiences including the specific services students used to seek help with their study and migration journey to ensure that participants could not be indirectly identified and all data were securely stored in accordance with institutional ethical guidelines. Interviews were conducted in 2024–2025. Most interviews were conducted in English, while some were conducted in Vietnamese to accommodate participants’ preferences, with three team members fluent in Vietnamese assisting. All interviews were transcribed verbatim.

**Table 1.** Demographics of the participants

No	Pseudonyms	Gender	Nationality	Study program	Age
1	Ping	Female	Chinese	Master of Teaching (Early Childhood Education)	30
2	Wei	Female	Chinese	Master of Construction Management	25
3	Kai	Male	Chinese	Master of Architecture	24
4	Ling	Female	Chinese	Bachelor of Banking and Finance	21
5	Bo	Female	Chinese	Master of Education	23
6	Nhi	Female	Vietnamese	Bachelor of Early Childhood Education	20
7	Thuy	Female	Vietnamese	Bachelor of Accounting	19
8	Tien	Female	Vietnamese	Master of Architecture	35
9	Duc	Male	Vietnamese	Bachelor of Property and Real Estate	20
10	Long	Male	Vietnamese	Bachelor of Artificial Intelligence	21
11	Kiran	Male	Indian	Master of Business Analytics	26
12	Kavya	Female	Indian	Master of Business Analytics	40
13	Arjun	Male	Indian	Masters of International Relations	-
14	Rahul	Male	Indian	Bachelor in Civil Engineering	23
15	Nisha	Female	Indian/Chilean	Graduate Diploma in Early Childhood Education	25
16	Anh	Female	Vietnamese	Bachelor of International Relations and Human Right (minor in Philosophy)	24
17	Minh	Female	Vietnamese	Bachelor of Arts (Music)	27
18	Hoa	Female	Vietnamese	Bachelor of Occupational Therapy	21
19	Jun	Male	Chinese	Master of Business (Sport management)	26
20	Xin	Male	Chinese	Bachelor of Economics (Finance)	22
21	Yun	Female	Chinese	Master of Professional Accounting	32
22	Lei	Female	Chinese	Master of Sport Management	23
23	Xia	Female	Chinese	Master of Commerce	24
24	Rohit	Male	Indian	Masters of Engineering – Mechanical with Business	30
25	Priya	Female	Indian	Master of Business Analytics	28
26	Maya	Female	Indian	Master of Business Analytics	24
27	Simran	Male	Indian	Bachelor in Civil Engineering (HONOURS)	23
28	Yu	Female	Chinese	Master of Construction Management	25
29	Amit	Male	Indian	Master of IT	25
30	Vikram	Male	Indian	Bachelor of Science	20

Data analysis was conducted using NVivo version 12 through a collaborative process involving members of the research team. The process began with repeated readings of all transcripts to build familiarity with the data and to develop an initial codebook, informed by the theoretical frameworks of governmentality and affect theory, as well as key policy themes in Australia’s international education landscape. A deductive coding strategy was employed using pre-identified theoretical concepts, while remaining open to emergent patterns to ensure interpretive flexibility.

The constant comparison method (Lincoln & Guba, 1985) was applied to identify patterns within and across participant groups, paying particular attention to how experiences differed by nationality and institutional context. This comparative approach enabled the integration of empirical data with existing literature and theoretical constructs. Data saturation was determined when analysis of successive transcripts produced no new themes, indicating that the sample adequately captured the diversity of perspectives relevant to the research questions.

NVivo supported data management and enabled the visualisation of code relationships and thematic patterns across cases. The software’s query functions were used to explore code frequencies, co-occurrences, and intersections between empirical findings and theoretical frameworks. Due to space limitations, this article presents a selection of excerpts that either reflect frequently recurring themes or offer distinctive insights into the affective dimensions of students’ policy experiences.

The researchers’ experiences as former international students provide them with familiarity with the challenges and perspectives of international students. However, they did not personally experience the most recent policy changes as international students. Their critical reflexivity, developed through engagement in multiple research projects on international education, helped to mitigate potential ‘insider’ bias in both data collection and interpretation.

By applying the governmentality framework and affect theory discussed above as a theoretical lens, the analysis reveals how policy design and enactments manage not only students’ movements but also their emotions, aspirations, and perceived entitlement to belong. The next section will discuss the affective dimensions of Australia’s risk-tiered visa system and other policy changes.

## Results and Discussion of Findings

### The Risk-Tiered Visa System and Nationality-Based Deterrence

Participants from China and Vietnam consistently articulated a perception of being specifically targeted by Australia’s newly implemented risk-tiered student visa assessment framework under the Migration Strategy 2023. Rather than perceiving the process as a neutral regulatory mechanism, these students described it as a discriminatory infrastructure disproportionately disadvantaging applicants based on nationality. The analysis of data shows that such administrative differentiation not only imposed practical burdens such as prolonged processing times, opaque rejections, and increased financial costs but also inflicted significant emotional stress, diminished trust, and undermined their sense of inclusion and belonging in the host country.

Ping, a Chinese female student studying Master of Teaching, exemplifies how multiple risk assessment layers compound to create disadvantage even within Australia’s most elite institutional tier. When asked about her observations of visa processing experiences within Chinese student communities, she described the frustration of navigating what she perceived as discriminatory processing:

I understand, because during this 2 year you may change your decision. It's totally fine, but I don't think this can be one of the rejection reasons for the students because sometimes when I read the comments or news from social media like especially from the student from China, mainland. They will get a rejection of their visa, even though we got already efficient amount of money to study here. Prove that I have a lot of money, and then I'm genuine to study here. I also get the offer like everything but they still get rejected. I don't know why. If you already [have] the offer from the university, and you also have enough money to study here (Chinese, female, Master of Teaching in Early Childhood Education).

Ping’s experience shows how multiple risk assessment layers operate within Australia’s visa governance system. Her observations reveal how students face rejection despite meeting all formal requirements such as adequate finances, university offers, and genuine study intentions. The persistent question ‘I don’t know why’ highlights the opacity of decision-making processes that leave students without clear explanations for rejections. Her reference to social media discussions among ‘students from China, mainland’ demonstrates how rejection experiences circulate within Chinese student networks, creating collective awareness of nationality-specific processing patterns.

Ping’s case is particularly significant given her position within Australia’s institutional hierarchy. Her university is classified as a lower-risk institution under Australia’s evidence-based framework, which, according to Ministerial Direction 107, should theoretically give advantages in visa processing by prioritising applications based on both the risk level of education providers and the applicant’s country of citizenship. However, her extensive engagement with Chinese student communities reveals that even institutional advantage cannot shield students from nationality-based processing disparities. Ping’s observations cannot be attributed only to isolated cases but must be understood as reflecting how institutional hierarchies intersect with the implementation of risk-based governance under Ministerial Directions 107 and 111, which prioritise visa processing based on both institutional provider and applicant country risk classifications. These overlapping risk systems – national, institutional, and individual – create different experiences that separate students even within the same country of origin, producing what might be called a ‘hierarchy of disadvantage’ among Chinese applicants.

This intersectional discrimination mechanism reinforces Fenton-Smith and Gurney’s (2025) argument that Australia’s tiered visa assessment framework has transformed visa governance into a technology of risk surveillance, wherein merit-based assessment is supplanted by prescriptive filtering grounded in both nationality and institutional classification. This governance operates through what Robertson (2019) conceptualises as ‘strategic ambiguity’, leaving students navigating opaque decision-making processes, uncertain outcomes, and implicit hierarchies of belonging. Such governance strategies exemplify Bacchi and Goodwin’s (2016) conceptualisation of governmentality**,** a mode of power that functions through subtle forms of control rather than explicit enforcement mechanisms, creating self-regulating subjects who internalise institutional logics of risk and worthiness.

For example, Nhi vividly recounted repeated rejections:

I failed the visa three times to study in Australia. Because they still needed a financial demonstration or something like that back then. I need to ask my relatives in America to prove that we have the finance to study abroad (Vietnamese, female, Bachelor of Early Childhood Education).

Nhi’s experience exemplifies the disproportionate burden placed upon students from countries designated as ‘high-risk’. Vietnam was previously considered a high-risk (Assessment Level 3) country in Australia’s student visa risk framework and in the Department of Home Affairs’ 30 September 2025 update it was upgraded to a moderate-risk (Level 2) country. The education-migration nexus has evolved into an education–work–migration pathway characterised by uneven spacetimes – periods marked by waiting, deferral, and contingent legality (Tran, Bui et al., 2025). Students like Nhi are caught in an ‘obstacle race’ (Mezzadra & Neilson, 2012), where regulatory goalposts shift unpredictably, and criteria remain deliberately ambiguous.

Wei, a Chinese postgraduate student, highlighted how nationality-based visa discrimination extends into systemic disadvantage:

Obviously, you can see, we have a huge trend of things, even for their graduate students, they got a longer research compared to students holding the Chinese passport. I think it's kind of influencing [...] how to locate themselves after five years, after 10 years (Chinese, female, Master of Construction Management).

Wei’s account demonstrates ‘affective hierarchies’ embedded within policy enactment, where nationality becomes a primary determinant of perceived risk and suspicion. Such practices reveal how nationality actively shapes institutional behaviours, relational trust, and academic opportunities, reinforcing a racialised hierarchy under the guise of administrative objectivity (Li & Whitworth, 2016).

Importantly, participants themselves often contextualised their experiences within broader geopolitical frameworks, interpreting visa delays and rejections as reflective of Australia’s strained diplomatic relationships, notably with China. This framing underscores international students’ political agency and acute awareness of their precarious positioning within global migration governance (Tran et al., 2023). Rather than passive policy subjects, students actively interpret, challenge, and navigate the geopolitical undercurrents shaping their mobility and migration journeys.

Kiran, an Indian postgraduate student studying Master of Business Analytics, questioned the fundamental assumptions underlying visa assessment: ‘How do you test whether they are genuine students or not?’ (Indian, male, Master of Business Analytics). This questioning reflects broader concerns about the differential treatment experienced by students from different countries. As another participant observed, the Australian Government’s approach to declining visas from certain regions reveals patterns where ‘if there’s anyone whose address is of Punjab [...] they were declining these visas’, while students from other backgrounds face different processing experiences. What emerges is a visa regime increasingly shaped by racial and geopolitical factors rather than transparent, merit-based criteria. It also reflects the hierarchy of Australian university sector in which the research-intensive universities which have significant numbers of international students (some as high as 50%) but are considered to be ‘low risk’ with regard to fast tracking student visas. These universities also have the assets to protect against any rapid reduced number of international students (Blackmore, 2023).

### The Genuine Student Test as Bureaucratic Borderwork

Within the landscape of policy changes, the replacement of the Genuine Temporary Entrant (GTE) requirement with the Genuine Student Test (GST) emerged as a major source of stress, uncertainty and perceived inequity among participants. Officially presented under the Migration Strategy 2023 as a measure to ensure system integrity and exclude applicants with concealed migration intentions, the GST was often experienced by students as a deeply discretionary, unpredictable and racialised form of bureaucratic gatekeeping.

Tien, a Vietnamese graduate student, highlighted:

When I was considering going, there weren't many changes like that. I would be directly affected. When I came here, I was already in that situation, I had no other choice, I had to try other ways. When I was preparing my application, it was still the same as before, no changes, but when I came here, then it changed. I know Australia changes a lot, and it was truly a shock to me (Vietnamese, female, Master of Architecture).

The psychological impacts of navigating GST were profound. Kavya, an Indian student, discussed the emotional repercussions:

The Genuine Student Test feels like proving you're not a liar. So how is it solving the purpose of immigration. Even if you speak to a student, the student has already prepared the question. How is it solving the purpose of the entire policy of coming up with this kind of a setup. It's not [...] any difference you can always write [...] These are very obvious questions, and you can also answer for it. Stick it on the wall, and keep reading that. The whole process made me so anxious instead of excited about coming here (Indian, female, Master of Business Analytics).

Kavya’s reflections indicate that GST acts not merely as administrative scrutiny but as a technology of (not) belonging, shaping students’ affective and psychological relationships with Australia even before their arrival (Griffiths, 2017; Robertson, 2019). These pre-arrival experiences establish a formative context for interpreting their subsequent social inclusion or exclusion within Australian institutions and society.

These insights resonate with Hong et al.'s (2022) critique of Australia’s contradictory governance logic, embracing diversity rhetorically while practising exclusionary processes. For participants, the GST embodies this contradiction, upholding the notion of system integrity while functioning as a racialised sorting mechanism disproportionately burdening applicants from Vietnam and other lower-income nations. Participants displayed acute awareness and critique of policy inconsistencies, recognising differential treatments based on nationality. Duc succinctly summarised this dynamic: ‘If you’re from certain countries, you must prove more, wait longer, and expect less’ (Vietnamese, male, Bachelor of Property and Real Estate). This perception shows that international students critically interpret state logics, attuned to geopolitical dynamics that shape their migration trajectories.

To summarise, the Genuine Student Test underscores how migration governance produces inclusion and exclusion not merely through outcomes but through the emotional and procedural mechanisms of waiting, uncertainty, and self-doubt (Bacchi & Goodwin, 2016; Robertson, 2019). Such pre-arrival policy experiences are crucial in shaping students’ early interactions with Australia’s institutions, influencing their sense of belonging and trust. This analysis illuminates the invisible yet significant emotional, psychological, and financial costs inherent in Australia’s contemporary migration regime, necessitating further scholarly and policy attention.

### Post-Study Work Rights Changes Creating Temporal Bordering

The reduction in Post-Study Work Rights duration emerged prominently in interviews, profoundly reshaping participants’ relationships with Australian higher education and their post-study aspirations. Although officially justified as rational alignments of graduate outcomes with labour market demands, students interpreted these changes as exclusionary signals – a recalibration of their right to belong.

Participants’ narratives revealed complex negotiations involving aspiration, belonging, and practical adjustments to shortened post-graduation stays. The contraction of the previously more generous PSWR period into shorter durations was experienced as temporal bordering, a policy that imposes subtle yet powerful temporal constraints, shaping migrants’ futures and emotional experiences. These reductions, along with age caps, have reshaped students’ life trajectories, including delayed migration goals, postponed planning, and career compromises.

Duc, a Vietnamese student pursuing property and real estate, expressed disillusionment about permanent residency prospects:

Australia seemed to offer a pathway to staying longer, which influenced my decision. But in reality, staying in Australia permanently is now very difficult, almost impossible. (Vietnamese, male, Bachelor of Property and Real Estate)

Duc’s experience echoes Robertson and Runganaikaloo’s (2014) concept of temporalities of migration governance, where time serves as a regulatory tool influencing migrant agency. The shortened duration converts potential opportunities into urgent constraints, transforming settlement prospects into experiences of insecurity.

Similarly, Ping, a Chinese postgraduate student studying Master of Teaching, highlighted how PSWR reduction compounded field-specific constraints and broader policy inequities:

To be honest, I don't agree with this policy, and I feel is very unfair for the student. In Australia, I can see many people, they would like to continue their study after work because 2 years work, you may change your perspective. Maybe you may find you're another passion, right? If you limit the people from 485 visa change back to 500 visa. It's actually not good for the individual and also for Australia as well (Chinese, female, Master of Teaching in Early Childhood Education).

Ping’s account aligns with Marginson's (2024a) critique of international education’s neoliberal logic, framing students as economic consumers whose promised pathways are abruptly altered by opaque policy shifts. This undermines trust in institutional communications and national policy coherence.

Ling, a Chinese student, illuminated the emotional toll associated with compressed timelines:

I think I will be impact because before the change, I maybe I will think I just work in Australia after I graduate. But now I will think two years not enough to move to get a PR. When I graduate, maybe I will go back, things like that? Because two years it's too short, not enough to move (Chinese, female, Bachelor of Banking and Finance).

Ling’s reflection exemplifies the concept of affective governance in international education, highlighting how policy shapes students’ emotional and psychological experiences. The repeated emphasis on ‘two years not enough’ reflects the psychological impact of temporal bordering, where policy changes transform previously viable pathways into sources of stress and uncertainty. Students are compelled to recalibrate their aspirations continually, feeling obliged to express gratitude and suppress frustration despite sensing systemic disposability. The psychological implications of these policies varied, affecting interpersonal relationships in complex ways.

The stark differences in treatment based on nationality were evident. Kiran, an Indian student pursuing Business Analytics, reflected on nationality-based advantages in post-study entitlements:

When I came last year, my visa was still like 5 years, 2 plus 5. It has been reduced to 3 years now... I'm lucky that I'm from India because all other countries have 2 years but I'm being from India, I have 3 years due to the good positive relationship between both the countries (Indian, male, Master of Business Analytics).

Arjun, another Indian student, described guilt over his relatively privileged position compared to peers of different nationalities, while others reported anxiety and isolation due to compressed future timelines. These emotional responses reveal deeper policy implications beneath ostensibly administrative adjustments. These experiences underscore visa durations as symbolic borders, structuring unequal access, rights, and aspirations. PSWR reductions compress students’ imagined futures, shaping and reshaping their identities and their experiences of societal inclusion (Soltani & Tran, 2021).

Participants demonstrated critical awareness of contradictions between institutional/agent marketing rhetoric and evolving policy constraints. Many expressed frustration with perceived ‘bait-and-switch’, where institutional narratives promise pathways that are increasingly blocked by unpredictable and frequent policy changes. Such discrepancies between marketing and reality generate significant credibility gaps within international student communities. Far from passive recipients of policy, students like Duc and Ping expressed feelings of betrayal because their expectations were accurately informed by previous policy contexts. Their responses indicate an acute understanding of shifting migration regimes and highlight the inequities embedded within nationality-based policy stratifications. Therefore, PSWR adjustments exemplify differentiated inclusion mechanisms shaped by geopolitical and economic considerations. These stratifications influence not merely practical mobility opportunities but also internalised perceptions of legitimacy and self-worth. Post-study visa durations emerge as powerful symbolic indicators, delineating who is encouraged to integrate into Australian society and who is implicitly directed to merely study, spend, and depart.

### Age Caps and Visa Hopping Bans as Temporal Disruption

Participants consistently identified the newly introduced age cap on Temporary Graduate visas and the ban on ‘visa hopping’ as policy mechanisms that created not only practical limitations but also profound emotional and psychological consequences. Introduced as part of Australia’s recent migration recalibration (Australian Government, 2024; Department of Home Affairs, 2025), these changes were experienced not as isolated regulatory updates, but as emblematic of a broader withdrawal of welcome, a temporal closing of Australia’s migration promise (Marginson, 2024a).

Tien, a Vietnamese graduate student pursuing architecture, explained how policy changes compressed career planning timelines:

I thought that after finishing my studies, with the 485 visa I would have time to work, and my plan would remain the same. Currently, I work part-time and according to my work situation, I basically have to prepare documents to submit my application before I even graduate (Vietnamese, female, Master of Architecture).

Tien’s experience reveals how shortened visa durations force students to prepare post-study work and immigration applications while still completing their studies, creating temporal pressure that transforms gradual transitions into overlapping processed. Other participants described how the visa hopping ban, preventing sequential enrolments or staggered migration strategies**,** disrupted carefully curated life plans. Bo, a Chinese Master of Education student in her final semester, recounted:

I think will impact my decision. I just imagine that after I finish my master degree, if I have any chance to have a work here, I will change my visa, maybe to working visa, right? But when I started to work, I have signed another source, if I have opportunities to do PhD or I want to do a second master, maybe I were back to student visa, right? If I want to back to student with Visa, I need to back to China and apply again, pay again. It's tricky, right? Because it costs a lot of our energy, it's so tricky (Chinese, female, Master of Education).

This rigidification of migration pathways constrains students’ imagined futures, which are shaped through the dynamic interplay of their plans, aspirations, identities and the structural realities they navigate (Soltani & Tomlinson, 2024; Soltani & Tran, 2021). Bo’s repeated emphasis on the process being ‘tricky’ captures how offshore application requirements (‘back to China and apply again, pay again’) transform seamless career progression into fragmented and costly processes. Bacchi and Goodwin (2016) emphasise that policy does not only regulate behaviour, but governs anticipation, shaping what feels imaginable and worth pursuing. In this context, the closure of sequential educational mobility – a once-viable strategy for skill development and extended stay – signals premature exit over long-term integration (Mezzadra & Neilson, 2012).

The emotional toll was palpable. Long, a Vietnamese student, recounted his shifting aspirations amid family pressures and policy uncertainty:

Well, it's a funny story because she's the opposite of other moms, other parents. She wants me to stay, get a job, get visa, permanent visa. It's just against my plan. I wanted to go back to Vietnam [...] I'm still deciding (Vietnamese, male, Bachelor of Artificial Intelligence).

Long’s statement reflects how temporal bordering and anticipatory governance (Griffiths, 2017) induce ongoing anxiety. His reoriented trajectory, from settlement to extraction, is a survival strategy in response to chronic policy uncertainty. These disruptions reveal how state practices recalibrate not just opportunities, but affective attachments to place.

Rahul, an Indian engineering student, described the cumulative effect of these shifts:

The increase of the visa fee more than double, it was a very harsh decision, I would say, from the government, it is a very big decision that would make people think that Australian government, it's not pro students, and they don't want students to be coming here [...] if you compare Australia to any of the other countries, the visa fee in Australia is the highest (Indian, male, Bachelor in Civil Engineering).

Rahul’s response underscores the affective dimensions of migration governance. Policies not only restrict movement but generate fear, instability, and doubt (Bacchi & Goodwin, 2016). Temporal constraints thus function as existential pressures that constrain how students plan, act, and imagine their futures (Tedeschi & Gadd, 2021).

Such experiences reveal deep tensions between policy rhetoric about attracting global talent and the lived realities of students whose aspirations are systematically compressed. For the students participating in this study, age caps and visa hopping bans are more than administrative rules – they discipline students into adjusting their ambitions downward, shifting their orientation from long-term integration to short-term compliance. These dynamics represent a subtle but potent form of governmentality, wherein students internalise exclusionary logics, engaging in self-regulation and quiet withdrawal (Hirschl & Shachar, 2019).

These policy effects reverberate beyond individuals, destabilising the broader ecosystem of international education. Hong et al. (2022) critique the structural economisation of international education. Our data complements this by showing how policies affect the emotional geographies of student life. As Collins (2021) notes, migration governance must be understood through its temporal as well as spatial articulations. International students face not only the absence of permanent residency pathways, but also the erosion of temporal security – what Robertson (2019) terms temporal precarity.

Beyond the administrative, the governance of time disrupts confidence, increases emotional stress, and severs students’ ability to plan, affecting familial relationships and long-term aspirations (Rung, 2017). These temporal constraints become invisible borders, determining not just when students must leave, but how they experience the present and envision the future. Nonetheless, student agency persists. Participants actively recalibrated strategies, from pursuing alternate migration destinations such as Canada to redefining post-study goals in Vietnam. This disrupts simplified portrayals of international students as economic agents, revealing them as complex navigators of structural constraints.

In essence, age caps for PSWR and visa hopping bans operate as stratified technologies of exclusion. They compress dreams, defer belonging, and signal Australia’s evolving stance on who may stay, settle, and contribute. These policies not only circumscribe student action but subtly reshape identity and aspiration, converting what began as educational mobility into a state of enduring uncertainty and premature departure.

### Fee Increases Leading to Disenchantment and Commodification

Significant increases in international student visa application fees emerged as a central concern across participant interviews. Students interpreted these changes not as isolated administrative adjustments but as part of a broader, extractive policy logic. In this regime, financial contributions are increasingly demanded without the guarantee of rights, certainty, or institutional reciprocity. Rather than viewing Australia’s international education as a transformative opportunity, many participants articulated a growing sense of disenchantment marked by economic exloitation and symbolic exclusion.

Nhi, a Vietnamese early childhood education student, captured this disillusionment about the commercialised nature of international education:

I didn't want to choose University A at first, not at all. But the agents, I think they were trying to reach the KPI for University A. They wanted their students to study in University A because half of my friends did the service through that agent went to University A (Vietnamese, female, Bachelor of Early Childhood Education).

Nhi’s reflections point to the commodification of international education, where students experience their roles not as learners or contributors but as revenue streams (Lomer, 2018). The affective toll of being positioned primarily as customers in a supposedly altruistic sector was a recurring theme, deepening students’ sense of alienation.

Bo, a Chinese postgraduate student, similarly expressed frustration about escalating costs and limited migration pathways:

Too much. I just think that if I back to my time to time to choose the uni, if the visa fee is so high, I won't choose Australia. I am torn between [a New Zealand university] and [a Go8 university]. I applied the two together, and [the Go8 university] gave me the offer firstly. So, I choose [the Go8 university]. Sometimes, I have a little bit regret. I sometimes think about the PR, because I choose [the Go8 university], but my major is not for immigrant, so I don't have any opportunity to get the PR (Chinese, female, Master of Education).

This dissonance between policy and discourse underscores what Hong et al. (2022) mentioned as a space where inclusivity is publicly celebrated but undermined in reality. For many students, increasing financial burdens paired with shrinking migration pathways rendered the entire system transactional and unstable.

Tien, who enrolled in a regional university as an aspirational compromise to boost her migration prospects, described feeling misled about policy stability:

The agent [...] shared why we should come to Tasmania to study instead of another state. They would provide a pathway for my family [...] Before coming, I didn't think it would change like that. I didn't think a country's government could change like that [...] A policy isn't something that can be changed just by saying it will change… (Vietnamese, female, Master of Architecture).

Tien’s experience reflects broader concerns about the fragility of policy promises and the mismatch between marketed incentives and shifting regulations (Stayner, 2019). The promised benefits of regionalisation promoted through the University Accord are increasingly seen as unstable and extractive rather than mutually beneficial.

The metaphor of students as ‘cash cows’ surfaced repeatedly. Arjun, an Indian student, explained the cumulative financial burden:

Maybe the tuition fee that is charged by international students is too much, pretty high [...] as an international student you have a lot of responsibilities and you have to take care of a lot of things at back home and here as well, and with the cost of living rising every day. So the fee could be managed or curtailed to an extent (Indian, male, Masters of International Relations).

Arjun’s remarks illustrate the erosion of trust as institutional and governmental justifications fail to align with lived realities. This aligns with critiques of neoliberal governance, where individuals are burdened with costs and risks while excluded from substantive entitlements. Importantly, students demonstrated critical reflexivity. Across interviews, participants described engaging in policy sense-making, sharing information, strategies, and warnings through peer networks, migration agents, and digital communities (Rung, 2017). Far from passive, they critically analysed state behaviour and its impacts on their futures.

This growing disenchantment carries reputational risks for Australia’s education sector. Participants described actively discouraging friends and family from choosing Australia due to high costs and unreliable post-study pathways. As Tien reflected in an interview: ‘It puts students in a very insecure situation, because we don’t know what tomorrow will bring’ (Vietnamese, female, Master of Architecture). Such sentiments reflect a broader relational rupture where the loss of trust in Australian institutions is not merely financial but existential. This shift aligns with critiques of neoliberal migration governance, where international students bear the risks of marketised education systems while being excluded from long-term protections or benefits (Collins, 2021; Hirschl & Shachar, 2019).

Students’ responses also reflect strategic adaptation. Rather than accepting commodification, they reframe themselves as exploited yet knowledgeable actors navigating pathways within – and increasingly beyond – Australia. Transnational digital platforms amplify these critiques, reshaping perceptions of Australia’s global standing in education markets (Choudaha, 2017).

All in all, visa fee increases serve as more than economic burdens; they are symbolic acts signalling the erosion of welcome. As profitability becomes the primary axis of value, students recalibrate their sense of belonging and make migration decisions accordingly. These dynamics have consequences that extend beyond the individual, reshaping mobility patterns, institutional trust, and Australia’s competitive edge in the global education landscape as they create affective environments of mistrust.

## Conclusion

Australia’s international education and migration policies have undergone significant recalibration since 2021, revealing a fundamental shift from inclusive opportunity to strategic differentiation. This study has critically examined how these reforms are not only implemented administratively but also deeply lived, interpreted, and evaluated by international students from Vietnam, China, and India. By conceptualising migration policy as a form of soft deterrence (Bacchi & Goodwin, 2016), this research foregrounds how mobility is governed through policy ambiguity, emotional regulation, and temporal disruption, rather than overt exclusion.

This research generates several compelling findings and original contributions to current understandings of the nexus of international education, migration and geopolitics in Australia.

The study identifies a hierarchical and stratified system of selective inclusion. It demonstrates that students’ experiences are not only differentiated by discipline and region but also significantly shaped by nationality. Indian students benefit from favourable bilateral arrangements, such as trade-linked PSWR provisions (Australian Government, 2024), which facilitate smoother post-study transitions. However, Chinese students reveal they often encounter intensified scrutiny and more constrained post-study pathways, compared to their Indian peers. These differences reflect broader geopolitical considerations that shape migration and higher education policies, influencing how students from different national contexts navigate their futures in Australia. This finding provides empirical extension to Shachar’s (2006) theory of stratified migration, revealing how geopolitical worth mediates inclusion, not only through national policy, but through the micro-level lived experiences of students. It also maps onto the stratified university sector in which some universities are more at risk than others.

The findings critically interrogate the limitations of regionalisation. Students often viewed regional enrolment as a strategic compromise, not a long-term commitment, thereby challenging policy assumptions around integration and place-based retention (Stayner, 2019). The study introduces the concept of ‘aspirational compromise’ as a new theoretical contribution. By illustrating how students strategically sacrifice educational aspirations or location preferences for perceived migration advantages in response to policy volatility, the concept ‘aspirational compromise’ challenges dominant framings of students as passive consumers (Choudaha, 2017) and aligns with Marginson's (2024b) depiction of students as agents navigating precarious terrains. This contribution enriches the migration literature by highlighting the active, strategic, and affectively mediated ways students respond to structural constraints.

Most significantly, the research advances the theorisation of migration governance by illustrating how policy operates affectively and temporally. Drawing on Bacchi and Goodwin’s (2016) governmentality framework and affect theory (Sedgwick, 2003), the findings reveal how affective governance, through visa uncertainty, strategic ambiguity, and suspicion, produces an internalised sense of illegitimacy among students. Chinese students’ accounts of performative ‘genuineness’ exemplify the burdens of emotional labour under suspicion. Meanwhile, the concept of ‘temporal bordering’ emerges as an original analytical lens through which to understand how policies such as age caps and reduced PSWR durations discipline future planning, limit aspirations, and induce chronic uncertainty (Robertson, 2019).

Based on our findings, several steps could help mitigate the affective harms experienced by international students and support the sustainability of Australia’s international education. First, enhancing transparency around visa criteria and application processes could reduce uncertainty and stress. Second, improving communication of policy changes between government, institutions and students is essential. Third, establishing student advisory panels would allow policymakers and universities to incorporate students’ lived experiences into program design, ensuring that policies better align with their aspirations and needs. Fourth, the increasing gap between Australia’s promotional narratives and students’ lived experiences undermines institutional credibility and long-term attractiveness. The long-standing narrative of opportunity is increasingly displaced by narratives of precarity and disappointment. Fifth, while regional policies may succeed in redirecting enrolments, their longer-term impact depends on investments in local labour markets, infrastructures, and support services. Without these, regional settlement will remain transient rather than transformative, exacerbating rather than resolving metropolitan concentration. Sixth, the increasingly transactional fee regime, marked by steep hikes without commensurate improvements in transparency or service, risks fuelling perceptions of extractivism. Participants described this as symbolic of Australia’s shifting stance from educational partner to profit-driven gatekeeper. Such perceptions jeopardise not only student wellbeing but also Australia’s diplomatic ties, particularly within the Indo-Pacific region.

There are several limitations of this study. The findings are based on a sample of 30 participants, which limits their generalisability to broader international student populations. Also, the use of snowball recruitment introduces potential selection bias, as participants may share similar networks or experiences. Although translation was carefully managed, there remains a risk of subtle meaning loss when translating the interviews in Vietnamese into English.

## Supplemental Material


Supplemental Material - Policy as Soft Deterrence: Impact of Policy Changes on International Students in Australia
Supplemental Material for Policy as Soft Deterrence: Impact of Policy Changes on International Students in Australia by Ly Thi Tran, Trang Le, Jill Blackmore, Baogang He, and Quan Vu in Evaluation Review.
