# Supplementary material for: Policy as soft deterrence: Impact of recent policy changes on international students in Australia
Source: Eval Rev. 2025 Dec 11;50(4):543–76. doi: 10.1177/0193841X251405523 (PMC13180022; doi:10.1177/0193841X251405523)
Supplement: Supplemental Material - Policy as Soft Deterrence: Impact of Policy Changes on International Students in Australia [file sj-pdf-1-erx-10.1177_0193841X251405523.pdf]

Appendix 1: Timeline of major policy changes in Australian international education (2021–2025)

November 2021: Launch of Australian Strategy for International Education 2021–2030

- Aimed at rebuilding the sector post-COVID-19.
- Four key priorities:
    1. Diversification of source countries
    2. Alignment with workforce needs
    3. Improved student experience
    4. Enhanced global competitiveness

December 2023: Ministerial Direction 107 issued

- Established visa processing priorities based on provider risk levels and applicants' country of citizenship.
- Criticised for disproportionately disadvantaging regional universities and certain source countries.

March 2024: Introduction of Genuine Student Test (GST)

- Replaced the Genuine Temporary Entrant (GTE) requirement for Student visa (Subclass 500).
- Students now respond to targeted questions instead of submitting a 300-word statement, supported by documentation.

July 2024: Comprehensive visa and enrolment reforms

- International student visa fee increased from $710 to $1,600.
- Temporary Graduate visa changes: maximum age reduced from 50 to 35 (except HDR students) and reduced PSWR.
- Indicative national enrolment cap set at 270,000 new students (145,000 for universities).
- Individual enrolment caps applied to each university.

July 2025: Visa fee increase

- International student visa fee further increased to $2,000.

December 2024: Ministerial Direction 111 introduced

- Delayed visa processing for institutions nearing enrolment caps, effectively acting as an informal cap.
- Contributed to declines in student visa applications and redundancies in regional universities.

August 2025: National Planning Level for 2026 announced

- Cap set at 295,000 international student places, 25,000 above 2025 levels.

October 2025: 2026 public university international student allocations announced

- 2026 international student allocations recognise universities demonstrating genuine focus on South-east Asia in line with Australia's national interest.

November 2025: Ministerial Direction 115 introduced

- Incorporated recent new overseas student commencements (NOSC) exemptions for public providers, including expanded PBSA accommodation, stronger Southeast Asia engagement and transnational education (TNE) delivery.

- Introduced a stricter control mechanism: providers cannot exceed 115% of their NOSC allocation, with those exceeding the threshold moved into a Priority 3 visa processing category.
